# Methylglyoxal: An Emerging Signaling Molecule in Plant Abiotic Stress Responses and Tolerance

**DOI:** 10.3389/fpls.2016.01341

**Published:** 2016-09-13

**Authors:** Tahsina S. Hoque, Mohammad A. Hossain, Mohammad G. Mostofa, David J. Burritt, Masayuki Fujita, Lam-Son P. Tran

**Affiliations:** ^1^Department of Soil Science, Bangladesh Agricultural UniversityMymensingh, Bangladesh; ^2^Department of Genetics and Plant Breeding, Bangladesh Agricultural UniversityMymensingh, Bangladesh; ^3^Department of Biochemistry and Molecular Biology, Bangabandhu Sheikh Mujibur Rahman Agricultural UniversityGazipur, Bangladesh; ^4^Department of Botany, University of OtagoDunedin, New Zealand; ^5^Laboratory of Plant Stress Responses, Department of Applied Biological Science, Faculty of Agriculture, Kagawa UniversityKagawa, Japan; ^6^Plant Abiotic Stress Research Group & Faculty of Applied Sciences, Ton Duc Thang UniversityHo Chi Minh City, Vietnam; ^7^Signaling Pathway Research Unit, RIKEN Center for Sustainable Resource ScienceYokohama, Japan

**Keywords:** abiotic stress, glyoxalases, methylglyoxal, reactive oxygen species, signaling crosstalk, stress tolerance mechanism

## Abstract

The oxygenated short aldehyde methylglyoxal (MG) is produced in plants as a by-product of a number of metabolic reactions, including elimination of phosphate groups from glycolysis intermediates dihydroxyacetone phosphate and glyceraldehyde 3-phosphate. MG is mostly detoxified by the combined actions of the enzymes glyoxalase I and glyoxalase II that together with glutathione make up the glyoxalase system. Under normal growth conditions, basal levels of MG remain low in plants; however, when plants are exposed to abiotic stress, MG can accumulate to much higher levels. Stress-induced MG functions as a toxic molecule, inhibiting different developmental processes, including seed germination, photosynthesis and root growth, whereas MG, at low levels, acts as an important signaling molecule, involved in regulating diverse events, such as cell proliferation and survival, control of the redox status of cells, and many other aspects of general metabolism and cellular homeostases. MG can modulate plant stress responses by regulating stomatal opening and closure, the production of reactive oxygen species, cytosolic calcium ion concentrations, the activation of inward rectifying potassium channels and the expression of many stress-responsive genes. MG appears to play important roles in signal transduction by transmitting and amplifying cellular signals and functions that promote adaptation of plants growing under adverse environmental conditions. Thus, MG is now considered as a potential biochemical marker for plant abiotic stress tolerance, and is receiving considerable attention by the scientific community. In this review, we will summarize recent findings regarding MG metabolism in plants under abiotic stress, and evaluate the concept of MG signaling. In addition, we will demonstrate the importance of giving consideration to MG metabolism and the glyoxalase system, when investigating plant adaptation and responses to various environmental stresses.

## Introduction

Most plants live in environments where they are constantly exposed to one or combinations of various abiotic stressors, such as extreme temperatures, salinity, drought, and excessive light, which can severely limit plant growth and development. For many important crop plants, exposure to stress(es) ultimately results in a considerable reduction in potential yields (Atkinson and Urwin, 2012). The interaction between abiotic stressors and plants is complex, eliciting multiple morphological, physiological, biochemical and molecular changes that can ultimately result in varying degrees of stress adaptation, enabling some plants to grow and develop under environmentally induced stress. Because of the number of metabolic pathways involved, and the compexity of their regulation, it is often difficult for researchers to identify the major regulatory components involved in the abiotic stress responses of plants (Sharma et al., 2013). Plants subjected to stress often produce toxic aldehydes (Hoque et al., 2012a,b,c; Hoque M.A. et al., 2012; Mano, 2012), of which methylglyoxal (CH_3_COCHO; MG) is the most ubiquitous. The reactive alpha-ketoaldehyde MG is cytotoxic to plant cells at high cellular concentrations, but it may act as an important signaling molecule at low concentrations (Yadav et al., 2005a,b; Singla-Pareek et al., 2006; Hossain et al., 2009; Kaur et al., 2015a,b). MG is produced in plant cells as a result of glycolysis, and its celluar concentrations are maintained at very low levels in the absence of any environmental stress (Kaur et al., 2015b). However, in response to abiotic stressors celluar concentrations of MG rapidly increase (Yadav et al., 2005a,b). Accumulation of MG can disrupt the normal functioning of cells, leading to alterations in metabolic behavior and, in some instances, the death of plants (Hossain et al., 2011). The glyoxalase pathway has evolved to enable plants, and other organisms, to withstand the detrimental effects of MG overproduction, by limiting the accumulation of MG in the cells under stress (Singla-Pareek et al., 2006, 2008; Alvarez Viveros et al., 2013). MG and the glyoxalases are now considered as potential markers for evaluating plant abiotic stress tolerance (Hossain et al., 2009; Kaur et al., 2014a,b,c; Nahar et al., 2015a). Although significant progress has been made in investigating MG metabolism and toxicity in plants, the role of MG as a signaling molecule in stress responses and the acquisition of stress tolerance in plants still remain unclear. In this review, we will summarize recent findings regarding MG metabolism and the glyoxalase system in plants under abiotic stress, evaluate the concept of MG signaling, and discuss the importance of MG metabolism in modulating plant abiotic stress responses and tolerance.

## MG Synthesis in Plants

**FIGURE 1:**
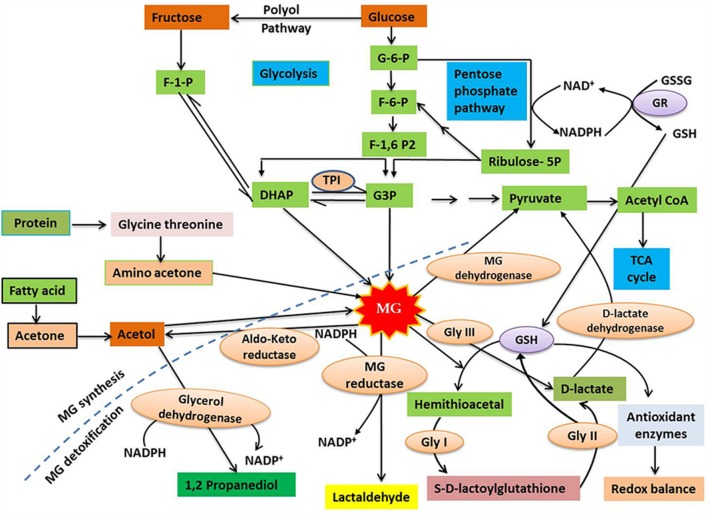
**A diagrammatic representation of methylglyoxal (MG) synthesis and detoxification in plants (modified from Ko et al., 2005; Hossain et al., 2011).** MG is primarily produced as a by-product of carbohydrate metabolism, with small amount produced during protein and lipid metabolism. Cytotoxic MG is efficiently degraded to form D-lactate by the action of the enzymes Gly I and Gly II, with the help of GSH. In addition, GSH-independent Gly III is able to convert MG to D-lactate. D-lactate dehydrogenase finally converts D-lactate into pyruvate, which enters TCA cycle via acetyl CoA. The broken line separates the synthesis and detoxification pathways of MG. For further discussion, see the text. Abbreviations are defined in the text.

In plant cells, the cytosol, chloroplasts and mitochondria are all considered to be potential sites of MG production. However, the specific rate and sites of MG production vary depending upon the cell or tissue type, the plant organ (e.g. leaves or roots), and the physiological state of the whole plant (Kaur et al., 2015a,b). Spontaneous production of MG occurs as a consequence of glycolysis, in metabolically active plant cells, from the reaction of the triose sugar phosphates glyceraldehyde-3-phosphate (G3P) and dihydroxyacetone phosphate (DHAP), both of which are photosynthetic intermediates (Yadav et al., 2005a; Takagi et al., 2014; Kaur et al., 2015a,b). This reaction is considered to be the principal route for MG formation under normal physiological conditions (**Figure 1**). Triose phosphates are unstable metabolites and show a high tendency to release an α-carbonyl proton, producing an enediolate phosphate intermediate that has a relatively low energy barrier for the elimination of phosphate groups (Richard, 1984). Thus, MG is formed by the deprotonation followed by the spontaneous β-elimination of the phosphate groups of triose phosphates (Richard, 1993). The enzymatic formation of MG occurs through the triose phosphate isomerase (TPI) that hydrolyzes G3P and DHAP, and removes phosphate to yield MG (Phillips and Thornalley, 1993). MG may also be formed by Amadori rearrangement during production of a Schiff base, which involves the reaction of the aldehyde groups of sugars with free amino acids or the amino acids of proteins (Vistoli et al., 2013). Other possible sources for MG formation include the auto-oxidation of surgars, as well as the metabolism of acetone and aminoacetone (Kalapos, 1999), although there is little evidence that these routes occur in plants.

## MG Detoxification in Plants via Glyoxalase and Other Metabolic Pathways

Methylglyoxal detoxification involves the conversion of MG to less toxic molecules, thus limiting its detrimental effects. The major route for MG detoxification in plants is the glyoxalase pathway, whose prescence was demonstrated in plants over 20 years ago (Norton et al., 1990; Maiti et al., 1997). In plant cells, the glyoxalase pathway is present in the cytosol and organelles, with high levels of glyoxalase enzyme activity found in chloroplasts and mitochondria (Yadav et al., 2008; Rabbani and Thornalley, 2012). There are two main enzymes associated with the glyoxalase pathway; glyoxalase I (Gly I; lactoylglutathione lyase; EC 4.4.1.5) and glyoxalase II (Gly II; hydroxyacylglutathione hydrolase; EC 3.1.2.6). These enzymes function in tandem to transform MG, and other 2-oxoaldehydes, to 2-hydroxyacids with the release of glutathione (GSH) (Thornalley, 1990). The detoxification of MG involves two irreversible reactions catalyzed by glyoxalases. The first step involves the reaction of MG with GSH, resulting in the formation of hemithioacetal that is then converted to *S*-D-lactoylglutathione (SLG) in a reaction catalyzed by Gly I. In the second step, which is catalyzed by Gly II, GSH is regenerated and D-lactate is formed by the hydrolysis of SLG (**Figure 1**). D-lactate, which is also considered a toxic compound if overaccumulated, is converted into pyruvate by D-lactate dehydrogenase (Engqvist et al., 2009; Wienstroer et al., 2012). Pyruvate, the major catabolic product of MG, can enter the tricarboxylic acid (TCA) cycle via acetyl CoA (**Figure 1**). The availability of cellular GSH is an important factor for MG detoxification via the glyoxalase system as the lack of GSH restricts hemithioacetal formation, resulting in MG accumulation (Hossain et al., 2011). Recently, a novel glyoxalase enzyme, named glyoxalase III (Gly III), was detected in plants, providing a shorter route for MG detoxification (Ghosh et al., 2016). Gly III contains a DJ-1/PfpI domain, and the presence of this domain has been used to confirm the existence of Gly III-like proteins in various plant species. Conventional glyoxalases (Gly I and Gly II) detoxify MG by converting it to D-lactate, with the help of GSH, but Gly III is able to irreversibly convert MG to D-lactate in a single step, without the need for GSH (**Figure 1**).

In addition to the glyoxalase system, several other pathways contribute to the detoxification of MG in plants. Other enzymes, including NADPH-dependent reductases, such as the aldo-keto reductases and aldehyde/aldose reductases, involved in detoxifying reactive carbonyls (Yamauchi et al., 2010), can reduce MG to the corresponding alcohol (Simpson et al., 2009; Narawongsanont et al., 2012). Another pathway is the irreversible oxidation of reactive aldehydes, including MG, to their corresponding carboxylic acids, which is catalyzed by aldehyde dehydrogenases (Kirch et al., 2005). However, the glyoxalase system is the most efficient MG detoxification system in plants under normal physiological conditions (Ghosh et al., 2016), and this pathway is very important for plants under stress (Singla-Pareek et al., 2006; Alvarez Viveros et al., 2013).

## MG Levels in Plants Under Stressful Conditions

Under normal metabolic conditions, plants usually maintain a lower level (30-75 μM) of MG (Yadav et al., 2005a; Hossain et al., 2009); however, an abrupt increase was observed in respone to abiotic stresses (Yadav et al., 2005a; Hossain et al., 2009; Mostofa et al., 2015a,b). Salt stress-induced inceases in MG levels were found in various plant species, including pumpkin (*Cucurbita maxima* L.) by 77%, tobacco (*Nicotiana tabacum* L., cv. BY-2) by 67% and potato (*Solanum tuberosum* L. cv. Taedong Valley) by 50%, compared with the respective controls (Hossain et al., 2009; Banu et al., 2010; Upadhyaya et al., 2011; Ghosh et al., 2014). Increased MG levels were also found in mung bean (*Vigna radiata* L.), *Lepidium sativum* and rice plants in response to drought (90–107%), and excessive Cd (60–260%) and Cu (106–156%) stresses, respectively, when compared with control counterparts (Nahar et al., 2015b; Mostofa et al., 2015b,c). These findings indicate that the increase in MG levels is a common response of plants to a variety of abiotic stressors, and that stress-induced MG could act as a generic signal molecule for plants under adverse environmental conditions.

## MG Toxicity in Plant Cells During Plant Growth and Development

In plant cells, MG accumulation has been shown to correlate with increased levels of intracellular oxidative stress, due to the enhanced reactive oxygen species (ROS) production (Maeta et al., 2005; Kalapos, 2008). MG accumulation may indirectly result in increased ROS production by decreasing available GSH levels and by impairing the function of antioxidant enzymes in plants under oxidative stress. In addition, MG can function as a Hill oxidant and catalyze the photoreduction of O_2_ to superoxide (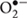) in photosystem I (PSI) (Saito et al., 2011). The production of 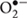 is deleterious as it can cause oxidative damage to cellular components.

Methylglyoxal is an α,β-dicarbonyl compound that can act both as a genotoxic and a glycation agent (Rabbani and Thornalley, 2014). MG has two functional groups; a ketone group and an aldehyde group, the latter being more reactive than the former (Leoncini, 1979). The dicarbonyl group within MG can readily react with the amine groups of proteins and nucleic acids, including DNA and RNA. The accumulation of MG is often called dicarbonyl stress, which has been implicated as a cause of tissue damage and aging (Rabbani and Thornalley, 2014). MG reacts with the amino acids lysine, cysteine and arginine producing glycated proteins, often referred to as advanced glycation end products (AGEs) (Ahmed and Thornalley, 2007), which can cause inactivation of proteins and oxidative damage to key cellular components (Thornalley, 2006). AGEs and dicarbonyl compounds, including MG, often accumulate in plant leaves upon exposure to high light or elevated CO_2_ concentrations (Qiu et al., 2008; Bechtold et al., 2009). Thus, it appears that the increase in sugar accumulation and changes in the metabolic flux of sugars, which occur at high CO_2_ concentrations, promote the production of MG and other reactive carbonyls, resulting in the accumulation of AGEs. In summary, excessive MG accumulation in plant cells under stress can inhibit cell proliferations, and cause the inactivation and/or degradation of proteins, inactivation of antioxidant defenses, leading to disruption of many cellular functions (Hoque et al., 2010; Hoque M.A. et al., 2012).

**FIGURE 2:**
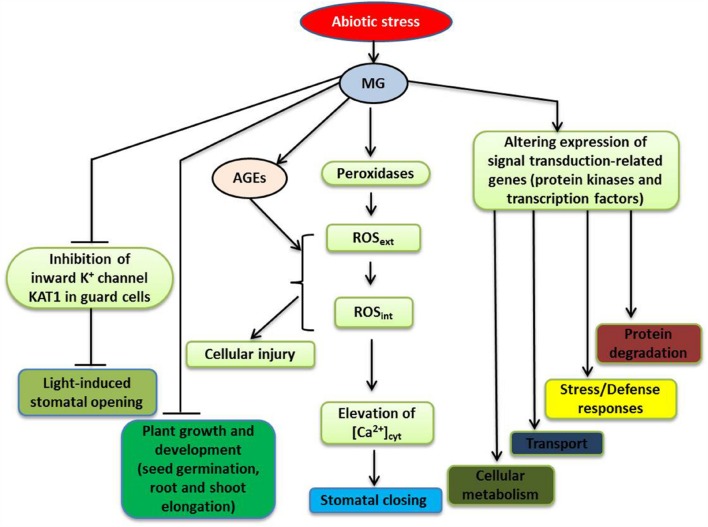
**A schematic model depicting the signaling roles of MG in plant during abiotic stresses (modified from Hoque et al., 2015; Kaur et al., 2015a).** Stress-induced MG participates in signal transduction by altering the expression of a number of genes, such as those encoding protein kinases and transcription factors (TFs), triggering various responses, including changes in general metabolism and ion/metabolite transport, stress and defense responses, as well as protein degradation. For further discussion, see the text. Abbreviations are defined in the text.

Indeed, MG showed toxicity to photosynthesis in the chloroplasts of spinach (*Spinacia oleracea* L.) (Mano et al., 2009), and the accumulation of MG in the *pdtpi* mutant, which lacks the plastid isoform of TPI, exhibited greatly reduced growth and increased chlorosis (Chen and Thelen, 2010). Yadav et al. (2005a) reported that accumulation of MG, as a result of salt stress, directly and adversely influenced plant developmental processes, such as seed germination and seedling growth, in tobacco plants. Similarly, Engqvist et al. (2009) found that *Arabidopsi*s plants grown on MS medium supplemented with MG (0.1 or 1 mM MG) exhibited a significant reduction in shoot and root growth. Later, the same group reported a dose-dependent decrease in root and shoot growth of *Arabidopsi*s, tomato and tobacco plants grown on MS medium containing 1 mM MG (Wienstroer et al., 2012). Furthermore, Hoque et al. (2012b) examined the inhibitory effects of MG on growth and development in *Arabidopsis* and suggested that 1 mM MG is toxic enough to significantly inhibit seed germination and root elongation in seedlings. However, concentrations lower than 0.1 mM MG had no influence on seed germination, but did reduced the rate of root elongation. In addition, concentrations of 1 mM MG or higher resulted in seedling chlorosis within 4 days of treatment. Recently, Kaur et al. (2015a) also reported that MG exposure caused a significant growth reduction in rice seedlings (*Oryza sativa* cv. IR4), with acute effects on root elongation in a concentration-dependent manner. The above findings highlight the growth inhibitory effects of MG on plants, and indicate that the MG levels causing toxic effects to plants vary depending on plant species, exposure time and perhaps age of plants.

## MG As A Signaling Molecule in Plants Under Stress

### MG-Induced ROS Regulation

In plants, ROS are primarily formed at low levels as metabolic by-products of photosynthesis and respiration in organelles through enzymatic reactions that take place in plant cell walls and the apoplastic space in response to pathogens (Ahmad et al., 2010; Sharma et al., 2012). In plants, the rates of ROS production dramatically increase under abiotic or biotic stress (Sharma et al., 2012), leading to the onset of oxidative stress. The enzymes involved directly in ROS production include plasmamembrane NAD(P)H oxidases, cell-wall peroxidases (Grant et al., 2000; Torres et al., 2002), apoplastic amine oxidases, oxalate oxidases and heme-containing peroxidases (Sewelam et al., 2016). Recently, Kaur et al. (2014a) reported that when MG levels in plant cells increased due to stress, ROS generation increased directly due to the presence of MG or indirectly due to the formation of AGEs. It has been reported that application of exogenous MG to tobacco plants, at concentrations between 0.5 and 10 mM, reduced the activities of antioxidant enzymes, such as glutathione *S*-transferase (GST) and ascorbate peroxidase (APX), leading to oxidative stress (Hoque et al., 2010; Hoque M.A. et al., 2012). In addition, Saito et al. (2011) also demonstrated that MG induced 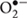 production in chloroplasts during photosynthesis. MG, at concentrations to 1 mM, has also been shown to cause a reversible induction of 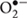 production in leaves of both wild-type *Arabidopsis* and NAD(P)H oxidase knock-out *atrbohD atrbohF* mutant plants, suggesting that salicylhydroxamic acid (SHAM)-sensitive peroxidases could be involved in this oxidative burst.

### Regulation of Stomatal Conductance Involving Cytosolic Ca^2+^ Oscillation and Inward K_in_ Channel Activation

Plants appear to have well-developed systems to sense and react to diverse environmental stimuli (Jia and Zhang, 2008). Stomata, which control CO_2_ uptake and minimize transpirational water loss, are capable of responding to various environmental stimuli, e.g., light levels, CO_2_ levels, temperature and humidity, and are often used as a model system to investigate cell-to-cell signaling in plants (Schroeder et al., 2001; Kim et al., 2010). Stomatal closure is an adaptive mechanism in plants, enabling them to survive in adverse environments (Osakabe et al., 2014). Stomatal closure is associated with increased concentrations of cytosolic calcium, [Ca^2+^]_cyt_, with oscillations in [Ca^2+^]_cyt_ occuring in guard cells in response to a diverse range of environmental stimuli (Pei et al., 2000; Mori et al., 2006; Young et al., 2006).

To investigate the mode of action of MG in stomatal guard cell signal transduction, Hoque et al. (2012a,c) investigated stomatal movement in *Arabidopsis* treated with different concentrations of MG. They found that at concentrations of MG up to 1 mM, MG behaved like a signal molecule as it induced stomatal closure, in a dose-dependant and reversible manner, without reducing the viability of guard cells. The induction of stomatal closure by MG involved an extracellular peroxidase-mediated oxidative burst and [Ca^2+^]_cyt_ oscillations (**Figure 2**). However, this MG-controlled induction did not require endogenous abscisic acid (ABA) nor endogenous methyl jasmonate (MeJA), and was not affected by deficiency in NAD(P)H oxidases. Thus, the studies of Hoque et al. (2012a,c) provided evidence that MG can induce stomatal closure, which is an important adaptive response of plants to environmentally induced stress.

Regulation of stomatal opening can greatly influence plant productivity and stress management (Dietrich et al., 2001), with inhibition of light-induced stomatal opening likely being occurred in plants under stress. The uptake of K^+^ into the guard cells accompanies light-induced stomatal opening, and inward-rectifying potassium (K_in_) channels play important roles in regulating K^+^ uptake (Schroeder et al., 2001). The K^+^ transporter of *Arabidopsis thaliana KAT1* gene is expressed in stomatal guard cells, and plants with a dominant negative mutation in this gene have reduction of K_in_ channel currents, which results in a reduced ability in regulating K^+^ ion flow and suppression of light-induced stomatal opening (Kwak et al., 2001). It has also been demonstrated that MG, in a concentration-dependent manner, can interfere with light-induced stomatal opening in *Arabidopsis*, and that this interference involves inhibition of K_in_ channel currents in guard cells, partially due to suppression of KAT1 channel activity (Hoque et al., 2012a,c) (**Figure 2**). According to Sato et al. (2009, 2010), protein kinase C (PKC) and stress-activated protein kinase SnRK2.6 (Snf1-related protein kinase 2.6) phosphorylate the C-terminal regions of KAT1, which modulates the activity of KAT1 channel. It is possible that MG can restrain the K_in_ channel activity by modifying C-terminal regions of KAT1, as well as other components, which inhibits stomatal opening.

### Expression of Stress-Responsive Genes in Co-ordination with ABA

Abiotic stresses, including drought, salinity and extreme temperatures, can induce the expression of many defense-related genes in plants. Stress-induced genes are important for plant survival as they encode proteins with both direct and indirect protective functions, and proteins that play important roles in signal transduction and gene regulation, both of which are important for coordinated stress responses (Ma et al., 2012; Thao and Tran, 2012; Yoshida et al., 2015). The plant hormone ABA is an important signal molecule for plant growth and development, as well as various physiological processes, including abiotic stress responses (Fujita et al., 2011, 2013; Osakabe et al., 2014). Many stress-inducible genes exhibit ABA-dependent gene expression patterns (Hadiarto and Tran, 2011; Todaka et al., 2015).

As ABA plays an important role in the integration of stress signals and downstream regulation of stress responses in plants (Hubbard et al., 2010; Weiner et al., 2010), it is possible that ABA could be involved in the responses that occur following MG accumulation. Hoque et al. (2012b) investigated the expression of the stress- and ABA-responsive genes *RD29B* and *RAB18* in *Arabidopsis* wild-type and ABA-deficient (*aba2-2*) mutant plants in response to MG treatment. They reported that MG significantly enhanced transcriptional levels of *RD29B* and *RAB18* in WT seedlings in a dose-dependent manner. In contrast, the transcription of neither *RD29B* nor *RAB18* was affected by MG in *aba2-2* mutant plants, indicating that ABA is involved in MG-induced up-regulation of *RD29B* and *RAB18* genes. This finding suggests that stress-induced MG may regulate stress-responsive genes in ABA-dependent pathway for plant adaptation to stress.

### MG-Responsive Signal Transduction Pathways

Plants have developed effective detection mechanisms and efficient signal transduction pathways to enable them to respond to various environmental stresses (Petrov et al., 2015). These pathways often involve multiple genes/proteins, operating in a coordinated manner, to regulate the expression patterns of the key genes, enabling plants to respond to a diverse range of external stimuli (Hadiarto and Tran, 2011; Gururani et al., 2015; Li and Tran, 2015). Kaur et al. (2015a) used microarray analysis to investigate gene expression profiles in rice exposed to exogenous MG, and study the molecular basis of MG responses. MG affected genes involved in hormone signaling, cell-to-cell communications, and chromatin remodeling. A number of genes encoding bZIP, MYB, NAC, WRKY, AP2/EREBP, and zinc finger transcription factors (TFs) were also found to be MG-responsive. In addition, various genes encoding protein kinases, including mitogen-activated protein kinases (MAP kinases), calcium/calmodulin-dependent protein kinases (CDPKs), Ser/Thr protein kinases, histidine kinases and receptor-like kinases, and OsRR2 type-A response regulator showed changes in their expression patterns. Since cellular MG levels increase in plants in response to stressful conditions, altered expression patterns of stress-inducible genes encoding TFs and protein kinases are expected to be observed following MG application (**Figure 2**). Using *in silico* analysis, Kaur et al. (2015a) identified conserved motifs as MG-responsive elements (MGREs) in the upstream regions of MG-responsive genes and provided the putative MGRE sequences (CTXXCTC and GGCGGCGX). The ability of MG to influence the stress-responsive signaling network highlights the importance of MG in plant stress responses.

## Glyoxalases in Plant Abiotic Stress Responses and Adaptation to Environmental Stressors

The glyoxalase system is involved in various cellular functions, but the involvement of this system in plant stress responses and tolerance is regarded as its most significant role (Kaur et al., 2014a). The glyoxalase system regulates MG levels in plants under stress and regenerates GSH. GSH and a high GSH/GSSG ratio are required to help protect plants against oxidative stress (Yadav et al., 2005a,b; Noctor et al., 2012), and GSH is directly or indirectly required for the functioning of various antioxidant enzymes, including GST, glutathione peroxidase (GPX), and APX (Yadav et al., 2008). Several studies have shown close links between the antioxidant and glyoxalase systems in plants, suggesting a direct influence of the glyoxalase system on ROS detoxification (Yadav et al., 2005a; El-Shabrawi et al., 2010; Upadhyaya et al., 2011; Mostofa et al., 2014a, 2015a).

An increase in glyoxalase enzyme activities occurs in plants in response to many different stressors, including osmotic stress, extremes of temperature, heavy metals and exposure to stress-related hormones, including MeJA, ABA and salicylic acid (SA) (Hossain and Fujita, 2009; Hossain et al., 2009). Transcriptomic and proteomic analyses of various plant species have improved our knowledge and understanding of the roles of glyoxalases in plant stress responses and tolerance (Singla-Pareek et al., 2003, 2006; Hossain et al., 2009; Lin et al., 2010; Mustafiz et al., 2011). Plant glyoxalase genes (*Gly I and Gly II*) have been cloned from various plant species and characterized in detail. The expression of *Gly I* and *Gly II* genes has been shown to be up-regulated in many plant species by a diverse range of environmental cues, and plants overexpressing either *Gly I* or *Gly II* showed enhanced plant abiotic stress tolerance (Singla-Pareek et al., 2003, 2006, 2008; Lin et al., 2010; Alvarez Viveros et al., 2013; Wu et al., 2013; Kaur et al., 2014a,c). The genetic manipulation of the glyoxalase system in plants has successfully contributed to improved tolerance to multiple abiotic stresses, such as salinity, heavy metals and MG treatments (**Table 1**). Transgenic plants overexpressing glyoxalase pathway genes have lower MG and ROS levels when exposed to stress, because they have better GSH homeostasis and retain better antioxidant enzyme functionality. Thus, glyoxalase enzyme levels can be used as phenomic biomarkers to indicate degrees of stress tolerance, and plants with high glyoxalase enzyme levels are potentially tolerant to a wide range of abiotic stresses (Kaur et al., 2014c). In **Table 1**, we summarized most of the successful transgenic studies that showed that transgenic plants, including important crop plants, overexpressing individually or together *Gly I* and *Gly II* have increased stress tolerance.

**Table 1:** Glyoxalase genes overexpressed in transgenic plants exhibiting enhanced abiotic stress tolerance.

Gene	Plant species	Response phenotype	Reference
*Gly I*	Tobacco (*Nicotiana tabacum)*	Improved salt stress tolerance	Veena et al., 1999
*Gly I*	Black gram (*Vigna mungo)*	Improved salt stress tolerance	Bhomkar et al., 2008
*Gly I*	*Arabidopsis thaliana*	Improved salt stress tolerance	Roy et al., 2008
*Gly I*	Rice (*Oryza sativa)*	Improved salt stress tolerance	Verma et al., 2005
*Gly I*	Tobacco (*Nicotiana tabacum)*	Improved salt stress tolerance	Yadav et al., 2005a
*Gly I*	Tobacco (*Nicotiana tabacum)*	Improved zinc tolerance	Lin et al., 2010
*Gly I*	Tobacco (*Nicotiana tabacum*	Improved tolerance to MG, salt stress, excessive mannitol and H_2_O_2_	Wu et al., 2013
*Gly I*	Tobacco (*Nicotiana tabacum)*	Improved tolerance to MG and salt stress	Mustafiz et al., 2014
*Gly II*	Rice (*Oryza sativa)*	Improved salinity tolerance	Singla-Pareek et al., 2008
*Gly II*	Mustard *(Brassica juncea)*	Improved salinity tolerance	Saxena et al., 2011
*Gly II*	Rice (*Oryza sativa)*	Improved salinity tolerance	Wani and Gosal, 2011
*Gly II*	*Arabidopsis thaliana*	Improved salt and anoxic stress tolerance	Devanathan et al., 2014
*Gly II*	Tobacco (*Nicotiana tabacum)*	Improved salinity tolerance	Ghosh et al., 2014
*Gly I* + *Gly II*	Tobacco (*Nicotiana tabacum)*	Improved salinity tolerance and set viable seeds under zinc-spiked soils	Singla-Pareek et al., 2003, 2006; Yadav et al., 2005b
*Gly I* + *Gly II*	Tomato (*Solanum lycopersicum)*	Improved salt stress tolerance	Alvarez Viveros et al., 2013
*Gly I* + *Gly II*	Carrizo citrange (*Citrus sinensis* × *Poncirus trifoliata)*	Improved salinity tolerance	Alvarez-Gerding et al., 2015


**Gly*, glyoxalase.*

In addition to the transgenic approach, alternative methods, such as treatments of seeds prior to sowing and/or plants with exogenous chemicals, e.g., plant growth regulators, osmoprotectants, signaling molecules etc., can also alter the glyoxalase system in plants, thereby improving stress tolerance (**Table 2**). For instance, treatment of rice seedlings with Ca has been shown to increase the activities of Gly I and Gly II, contributing to the reduction in As- and Cd- induced growth inhibition (Rahman et al., 2015a,b). Mostofa and Fujita (2013) reported that a SA pre-treatment of rice seedlings under Cu stress alleviated Cu-toxicity by increasing the capacity of both antioxidant and glyoxalase systems. **Table 2** lists most of the important studies in which chemical treatments were used to influence the glyoxalase system, leading to enhanced stress tolerance.

**Table 2:** Effects of exogenous chemicals on glyoxalase systems and abiotic stress tolerance.

Plant species	Types of stresses	Exogenous chemicals	Responses of glyoxalases (Gly I and II)	Concentration of MG	Reference
Rice (*Oryza sativa* L.)	As, Cd	Ca	Gly I ↑ Gly II ↑ (As)	↓	Rahman et al., 2015a,b
			Gly I ↑ Gly II ↑ (Cd)		
Rice (*Oryza sativa* L.)	Cu	SA	Gly I ↑	ND	Mostofa and Fujita, 2013; Mostofa et al., 2014a
			Gly II ↑		
Rice (*Oryza sativa*L.)	Heat	Spd	Gly I ↑	↓	Mostofa et al., 2014b
			Gly II ↑		
Rice (*Oryza sativa* L.)	NaCl, Cu	Tre	Gly I ↕ Gly II ↑ (NaCl)	↓	Mostofa et al., 2015a,b
			Gly I ↑ Gly II ↑ (Cu)		
Rice (*Oryza sativa* L.)	Cd, NaCl	H_2_S	Gly I ↓ Gly II ↑ (Cd)	↓	Mostofa et al., 2015c,d
			Gly I ↓ Gly II ↑ (NaCl)		
Mung bean (*Vigna radiata* L.)	Cd	Pro and GB	Gly I ↑	ND	Hossain et al., 2010
			Gly II ↑		
Mustard (*Brassica juncea*L.)	Drought	Pro and GB	Gly I ↕ Gly II ↑	ND	Hossain et al., 2014
Tea (*Camellia sinensis*L.)	Cold	Pro and GB	Gly I ↑	ND	Kumar and Yadav, 2009
			Gly II ↑		
Tobacco (*Nicotiana tabacum*L.)	NaCl	Pro and GB	Gly I ↑	↓	Hoque et al., 2008
			Gly II ↕		
Mung bean (*Vigna radiata* L.)	Heat, Drought	GSH	Gly I ↓ Gly II ↑ (Drought)	↓	Nahar et al., 2015b,c
			Gly I ↑ Gly II ↑ (Heat)		
Wheat (*Triticum aestivum*)	Heat, NaCl	NO	Gly I ↑ Gly II ↕ (Heat)	ND	Hasanuzzaman et al., 2011a, 2012b
			Gly I ↑ Gly II ↑ (NaCl)		
Rapeseed (*Brassica napus*)	Drought, NaCl, Cd	Se	Gly I ↑ Gly II ↑ (Drought)	ND	Hasanuzzaman and Fujita, 2011; Hasanuzzaman et al., 2011b, 2012a
			Gly I ↑ Gly II ↑ (NaCl)		
			Gly I ↑ Gly II ↑ (Cd)		
*Ficusconcinna*	Heat	BRs	Gly I ↑ Gly II ↑	↓	Jin et al., 2015


*As, Cd, Cu, Ca, SA, Spd, Tre, H_2_S, Pro, GB, GSH, NO, Se, and BRs correspond to arsenic, cadmium, copper, calcium, salicylic acid, spermidine, trehalose, hydrogen sulfide, proline, glycinebetaine, glutathione, nitric oxide, selenium, and brassinosteroids, respectively. Gly, glyoxalase; ↑, increased; ↕, unchanged; ↓, decreased; ND, not determined.*

## Conclusion and Future Perspectives

Recent studies of MG metabolism have revealed many important functions of MG related to stress responses and tolerance in plants. The excessive accumulation of MG in plants is an inevitable stress, but MG can stimulate the components of different stress-protection pathways (**Figure 2**; Engqvist et al., 2009; Hoque et al., 2012a,b,c; Wienstroer et al., 2012; Kaur et al., 2015a), which could be considered as an acclimation/adaptation process. The glyoxalase pathway scavenges MG and confers tolerance to multiple stresses; and thus, MG levels and glyoxalase pathway are closely associated with abiotic stress tolerance in plants. The signaling roles of MG in up-regulating stress-responsive pathways and its potential to active multiple pathways have made MG a suitable marker for abiotic stress tolerance in plants. Recent progress made by genome-wide and *in silico* analyses has revealed intricate regulatory networks associated with MG signaling, which control gene expression, protein modification and the metabolite composition of plants. Further omic studies investigating the roles of MG would be worthwhile to improve our understanding of multiple abiotic stress tolerance. In-depth understanding of the interactions of MG with Ca^2+^, ROS, NO, H_2_S, plant hormones, TFs, and the glyoxalase system, as well as with other MG detoxification systems in different subcellular compartments will reveal more regulatory roles for MG in plant abiotic stress responses and tolerance.

## Author Contributions

TH and MH conceived the idea. TH, MH, MM, DB, MF, and L-ST wrote the manuscript. All authors read and approved the final manuscript.

## Conflict of Interest Statement

The authors declare that the research was conducted in the absence of any commercial or financial relationships that could be construed as a potential conflict of interest.
